# GROW: a model for mentorship to advance women's leadership in global health

**DOI:** 10.1017/gheg.2018.5

**Published:** 2018-04-22

**Authors:** K. M. Yount, S. Miedema, K. H. Krause, C. J. Clark, J. S. Chen, C. del Rio

**Affiliations:** 1 Hubert Department of Global Health and Department of Sociology, Asa Griggs Candler Chair of Global Health, 1518 Clifton Rd. NE, Atlanta, GA 30322, USA; 2 Department of Sociology, Emory University, Atlanta, Georgia, USA; 3 Department of Behavioral Sciences and Health Education, Emory University, Atlanta, Georgia, USA; 4 Hubert Department of Global Health, Emory University, Atlanta, Georgia, USA

**Keywords:** Global health, Global Research for Women (GROW), mentorship, policy and society, women's empowerment, women's leadership

## Abstract

In this essay, we discuss the under-representation of women in leadership positions in global health (GH) and the importance of mentorship to advance women's standing in the field. We then describe the mentorship model of GROW, Global Research for Women. We describe the theoretical origins of the model and an adapted theory of change explaining how the GROW model for mentorship advances women's careers in GH. We present testimonials from a range of mentees who participated in a pilot of the GROW model since 2015. These mentees describe the capability-enhancing benefits of their mentorship experience with GROW. Thus, preliminary findings suggest that the GROW mentorship model is a promising strategy to build women's leadership in GH. We discuss supplemental strategies under consideration and next steps to assess the impact of GROW, providing the evidence to inform best practices for curricula elsewhere to build women's leadership in GH.

## Women's under-representation in global health leadership

Women are under-represented in leadership in global health (GH) across academic, governmental, and non-governmental institutions [1–4]. Across 191 countries, only 51 have a woman minister of health [5]. At Emory University, 91% of undergraduate minors and 84% of masters students in GH are women; yet, 75% of full professors (31 of 41) and 75% (six of eight) of named professors in GH are men. Women, including women of color, sexual minority women, indigenous women, other minority women groups, and women from lower income countries, remain disproportionately under-represented in leadership positions [6]. Women from different backgrounds face disparate barriers to leadership, which must be overcome to support the leadership potential of *all women* in GH [7].

## Rationale for women's leadership in GH

Women's representation in GH leadership matters on the simple grounds of justice. Empirically, women make up 50% of the world's population, bear the unique burden of certain causes of death [8], experience more years than men of life lost due to disability [9], comprise at least two-thirds of the GH workforce [5], and provide disproportionate unpaid care for the sick [5, 10]. Women contribute around US$3 trillion to GH care, but nearly half of this (2.4% of global gross domestic product) is unpaid [2]. Given women's unique needs and contributions, women should have equal formal, descriptive, and substantive representation in the ranks of GH leadership. Equality in *formal representation* means having the same opportunities as men to participate in leadership, without discrimination based on gender or other intersecting identities. Equality in *descriptive representation* means that women are represented in equal numbers in positions of leadership. Equality in *substantive representation* means that women's interests are advocated in decision-making circles. Emerging evidence suggests that women need to be in leadership to have their interests represented [11, 12].

## Call to action and role of women's mentorship networks

The recent inaugural Women Leaders in Global Health (WLGH) conference established principles to advance women's leadership in GH. Recommended strategies included increasing the visibility of women by ensuring gender balance in all spheres of academia, nominating and promoting women for important committees and awards, advocacy for a culture that values work-life integration, eliminating gender gaps in pay, cultivating thought leadership among women professionals, addressing data gaps that mask persistent gender disparities in leadership, and promoting accountability. One strategic priority of the WLGH Initiative includes mentorship for women's leadership in GH.

## GROW empowerment model for mentoring women leaders in global health

Given this ambitious agenda, models for mentorship are needed to position women for leadership in GH [3, 4]. Mentorship can be a powerful tool to enhance the capabilities of individual women and to strengthen their collective capabilities to advance in the ranks. Our focus on mentoring to enhance capabilities differs from the typical psychosocial and professional outcomes that feature in the mentoring literature [13, 14]. Enhancing individual *and* collective capabilities are essential to empower women for leadership in GH.

Here, we describe the theory of change and present pilot data for the mentorship model, Global Research for Women (GROW). GROW is an interdisciplinary, global initiative to catalyze empowerment, health, and freedom from violence for women and girls globally. Our guiding principle is that women's and girls’ empowerment is a pillar of sustainable development and is inextricably linked with their health and freedom from violence. Our strategic priorities are to advance scholarship, to cultivate leadership, and to generate dialogue that catalyzes social change through evidence-based policies, programs, and collective action for women's and girls’ empowerment.

**Fig. 1.**
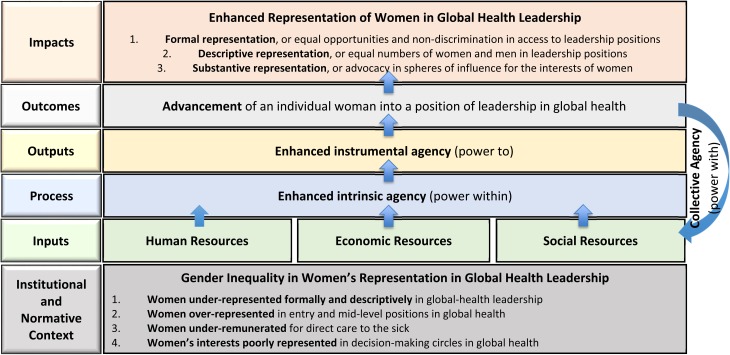
GROW empowerment model for mentorship and the advancement of women's leadership in global health.

Inclusive *with all* women and with men, the GROW mentorship model is adapted from a theory for women's empowerment developed by feminist economist, Naila Kabeer [15]. We define *women's empowerment in GH* as the process by which women acquire new *human*, *economic*, and *social resources*, which enable them to exercise *agency*, or *the capacity to make strategic life choices to enhance their professional development in a context in which these capabilities were once denied*. Below, we describe the components of this empowerment-based mentorship model to advance women's careers in GH (Fig. 1).

Expanding *human resources* involves advanced theoretical and methodological training enabling scholars to acquire new knowledge and skills in subfields of GH; opportunities to lead and to co-author publications with our global network of affiliates; in-country practicums to build field experience; guidance on how to build effective teams for research, practice, or advocacy; discussions on emotional intelligence, including skills to negotiate on one's own behalf and to manage professional conflicts; and training on ethical principles for robust work in GH.

Investing *economic resources* includes advocacy for undergraduate, graduate, and post-graduate scholarships; recommendations for prominent internal and external fellowships, awards, and positions; mentorship on grant writing; opportunities to participate on externally funded team projects at all stages of career; and financial support to attend and to present at professional conferences.

Expanding *social resources* includes integration into interdisciplinary, project-based teams and peer groups to build collaborations; exposure to formal and informal professional networks; opportunities to attend professional conferences; introductions to professionals with similar career interests; and in-country internships to deepen global networks. Social resources also include the amplification of accomplishments via the media, including press releases, webinars, newsletters (http://www.growemory.org/archives), blogs (http://www.growemory.org/growblog), and social-media posts.

*Agency* is an intermediate outcome that may arise from the above enabling resources. Agency encompasses an *intrinsic* belief in one's capabilities, the *strategic enactment* of one's aspirations or preferences, and the *collective action* of women to advance women's leadership in GH. *Intrinsic agency*, or power within, entails the development of self-confidence, self-efficacy, and a critical awareness of one's professional worth and potential contributions. *Instrumental agency*, or power to, entails the enactment of strategic, or meaningful, decisions that advance one's career. *Collective agency*, or power with, entails a group's shared belief in its joint capabilities to organize and to execute mutually agreed actions for shared achievements. More than the sum of individual agencies, collective agency emerges from the interactive, coordinated, and synergistic dynamics of the group [16]. Intrinsic and instrumental agency, as individual-level outcomes of mentoring, are rarely examined in mentoring models [13, 17]. Collective agency has never been conceptualized or measured as an outcome of network-based models for mentoring. Through these pathways, GROW leverages the achievements of women leaders in GH to benefit women at all career stages.

An overarching theme of GROW is *feminist praxis*. Research documenting the inequalities, injustices, and violence faced by women and girls, and advocacy for women's equality, has been met with scrutiny, and even backlash [18]. GROW trains scholars to develop new theory and to apply rigorous methods, producing exceptional evidence to meet this resistance and to inform debates that advance women's interests. GROW's mission to empower early career professionals to produce scholarship of exceptional quality necessitates robust, feminist approaches to the design, implementation, analysis, and dissemination of research [19]. Mentorship from senior GROW affiliates on feminist ethical guidelines enables scholars to balance participant autonomy and safety in each study of empowerment and violence. GROW's networks provide opportunities for training on disciplinary standards within this specialized field.

## Testimonials from GROW mentees

In Table 1, we present qualitative testimonials from graduates who received a pilot implementation of GROW since 2015. Qualitative evidence suggests that the focus on empowerment is attractive and capability enhancing. Trainees highlight the relevance of an interdisciplinary network supporting mutual growth, and rigorous research undergirded by a shared commitment to improve collectively the lives of women and girls. Excerpts from testimonials contextualize the capability-enhancing benefits realized by graduates mentored using the GROW framework. These testimonials reveal how GROW mentees have benefitted from the resources provided and the enhanced agency cultivated to advance their careers in GH. More information about the GROW network, access to scientific resources, and testimonials of the capability-enhancing benefits of GROW can be found at http://growemory.org/.

**Table 1.** Testimonials of the impact of GROW mentorship on the capabilities for leadership in global health

Domain of women's empowerment	Testimonial
Human resources	‘The GROW network is a fantastic place for like-minded scholars to collaborate and develop research. Many of the members of the GROW team have helped to foster a greater understanding of new methods of global research while encouraging thinking that will lead to impactful projects.’ ‘The website itself is a treasure trove of resources on women's health, gender empowerment, and especially relevant to me as an academic who studies gender-based violence, the breadth and depth of research in cutting-edge practice is an intellectually stimulating environment.’
Economic resources	‘I wanted to thank you again for your support in my attendance at the 2017 Society for the Scientific Study of Sexuality (SSSS) conference where I was able to co-present the continuation of my thesis work… It was a wonderful experience and I am very glad I had the opportunity to listen in on some very mind-opening seminars on sexual violence and sexual and reproductive health.’ ‘As a master's student and GROW member, I received a range of financial, scholastic, and professional support. To finance my summer practicum abroad, I worked closely with two senior members of GROW…[who] mentored me as I drafted a Global Field Experience (GFE) funding proposal, by sharing examples from their previous work, walking me through proposal norms in the public health discipline, helping me clarify my funding goals, and reviewing my proposal. Ultimately, I submitted a successful proposal that largely funded my practicum experience…I had funds to support my student job with GROW [and] these same GROW members submitted letters of recommendation on my behalf, including for my current position at a large international organization…’
Social resources	‘The mentorship I received, particularly through GROW network, encouraged me as public health professional to stay involved in research by build[ing] relationships that extended beyond my time at Rollins. The community deepened my understanding [of] what it means to belong to a research group by helping me to effectively build connections, share my own work, and exchange ideas in new and meaningful ways. These are all things that I have carried with me into my new role.’ ‘I think the way that GROW has influenced my work has been to connect me as a single, young, early career social scientist, with other more established people across this field for me to think through my research questions, to think through my research methods, to really critically analyze where my research is needed, how it's needed, and how I can go about implementing my ideas.’ ‘…you lead by example when you promote other women's work. You devote time to building up other researchers…in an academic environment that can otherwise be very cut-throat. I recall … a long talk … about taking ownership of my work … It was clear that that was part of a larger message that you were trying to convey about our role in academia, with its clear power dynamics. I am not sure how one would describe or classify that type of leadership, but I know that I have appreciated it!’
Intrinsic agency	‘I was better prepared for my current faculty position and felt more confidence going into this position (assistant professor at [University] department of Health Science) due to the mentoring and experiences I had…’ ‘Without GROW, it is unlikely I would have the depth and diversity of the hard and soft professional skills I use in my work now…My GROW work bolstered my professional skills and confidence.’
Instrumental agency	‘I was further benefitted by the opportunity to work with a team of colleagues on a variety of papers. Together we discussed the most important research questions to ask, problem solved challenges, and collaborated on completing projects. This environment prepared me for developing new collaborations and research projects…I also appreciated the informal discussions that I had…about negotiating faculty contracts and the added challenges that women sometimes face.’ ‘Further, my past GROW experiences shape the day-to-day choices I make about my current research – whether I am anticipating challenges enumerators might face in the field, structuring an interview guide, or mediating conflict between project partners.’
Collective agency	‘What I like about GROW is that it connects interdisciplinary scholars together, and we learn a lot from each other, whether it be global health, public health, nursing, medicine.’ ‘Being embedded in a network that fosters respectful, mutually beneficial cross-national and cross-cultural collaboration has amplified the impact of my work and my contribution to teams that share my commitment to women's health and empowerment.’
Career advancement	‘I just wanted to let you know that I've verbally accepted a tenure track offer with [University] to start next fall. Thank you for all of your support, writing a letter, and being on my committee. [University] has very much encouraged me to continue working with current colleagues and has been very impressed especially by the work from GROW. I certainly hope we can keep working together even as I look towards next steps.’ ‘As a master's student in GROW, I edited survey instruments and developed instruction manuals for data collection to enhance data quality. My finest achievement, however, was designing and conducting a qualitative study. The opportunities I had to undertake these tasks with careful, thoughtful mentorship from senior members of GROW empowered me to secure a great job post-graduation. During my job search, I felt comfortable matching my previous experiences with potential jobs’ qualifications, knowing I had the experience from GROW needed to excel as a future employee.’ ‘I feel fortunate that the study I was on as part of GROW was … quantitative and qualitative…My mixed-methods work with GROW taught me how to communicate what I … do as a qualitative researcher and helps me better understand my quantitative-based colleagues. That makes me a stronger colleague.’
Women's Leadership in Global Health	‘We did a research project on the aspirations of young women…on which [affiliate] of Emory and [affiliate of University] were PIs. I worked with the qualitative data and focused on…how gender norms in [country] are changing, and how being in the middle of that change presents a challenge for young women. On the one hand they're being told to get an education and that they can work. But on the other hand, the opportunities can be limited by social constraints about appropriate roles for women – and there's still a very strong expectation that they're supposed to be at home. There's a catch 22 where they want to have a career and have aspirations for one, but they can't make it work because they are expected to get married and have children and stay home to raise them. I got my article published relatively easily in a good journal and I'm very proud of it.’

Excerpts include testimonies from women and one man mentee of GROW.

## Next steps

Our pilot data suggest that the GROW model to build women's leadership in GH is promising. GROW strategies continue to evolve as we recognize, build up, and realize the potential for women's leadership in GH, inclusive of all women in this field. Our team is surveying masters-of-public-health and mid-career fellows alumni to assess, using the GROW model, (1) the human, economic, and social resources that men and women received during their usual training and (2) the associations of these resources with the measures of career-related intrinsic and instrumental agency and with acquiring a leadership position in GH. This survey will enable us to select additional content to further the leadership capabilities of women in GH. We expect that post-graduate leadership training and on-going career mentoring will be a promising supplemental strategy. If the survey findings corroborate our expectations, we will design a supplemental program and conduct a randomized-controlled trial to compare the empowerment processes and career trajectories of women graduates randomly selected for the program, *v*. women graduates randomly selected for usual training only and all male graduates in the same cohorts who receive usual training. A favorable causal impact of the program would guide best practices for all Departments of Global Health to modify and to supplement their graduate curricula in ways shown causally to enhance women's leadership in GH.
